# A single-center retrospective study on the clinical features of thyrotoxic periodic paralysis

**DOI:** 10.1371/journal.pone.0308076

**Published:** 2024-08-01

**Authors:** Kota Yamada, Akiyo Tanabe, Makiko Hashimoto, Mitsuru Ohsugi, Kohjiro Ueki, Hiroshi Kajio

**Affiliations:** 1 Department of Diabetes, Endocrinology and Metabolism, Center Hospital, National Center for Global Health and Medicine, Tokyo, Japan; 2 Department of Diabetes & Metabolic Diseases, Graduate School of Medicine, The University of Tokyo, Tokyo, Japan; 3 Department of Medical Examination Center, National Center for Global Health and Medicine, Tokyo, Japan; 4 Diabetes and Metabolism Information Center, Research Institute, National Center for Global Health and Medicine, Tokyo, Japan; 5 Diabetes Research Center, Research Institute, National Center for Global Health and Medicine, Tokyo, Japan; General Sir John Kotelawala Defence University Faculty of Medicine, SRI LANKA

## Abstract

**Purpose:**

Thyrotoxic periodic paralysis (TPP) is characterized by muscle paralysis and significant intracellular potassium movement resulting in hypokalemia. Since TPP is a rare condition, only a few studies have explicated the clinical characteristics of patients with this disease. This study aimed to elucidate the clinical characteristics of patients with TPP by comparing them with those with thyrotoxicosis without paralysis (non-TPP) and sporadic periodic paralysis (SPP).

**Methods:**

This was a single-center retrospective cohort study. Clinical data of patients with hyperthyroidism (n = 62) or periodic paralysis (n = 92) who were emergently admitted to our hospital was extracted from the electronic medical records and analyzed.

**Results:**

All patients in the TPP group (15 males and 2 females) had Graves’ disease, with 14 being newly diagnosed. The average serum potassium level on admission was 2.3±0.75 mEq/L. No significant correlation was observed among serum potassium level, amount of potassium required for normalization, and thyroid hormone levels. The TPP group showed significantly younger age, higher male ratio and body mass index (BMI), and lower serum potassium and phosphorus levels than the non-TPP group, which comprised 36 patients with Graves’ disease. No significant differences were observed between the TPP and SPP (n = 11) groups in terms of age, sex, BMI, serum electrolyte levels, potassium requirement for normalization, and recovery time.

**Main conclusions:**

Considering that most patients with TPP have undiagnosed Graves’ disease, distinguishing TPP from SPP based on clinical information and course alone is difficult in emergency settings. Therefore, for early detection and launch of specific treatment of Graves’ disease, screening for thyroid hormone and anti-thyroid stimulating hormone receptor antibody levels is necessary when treating patients with periodic paralysis.

## Introduction

Periodic paralysis is a rare condition caused by abnormal excitability of the sarcolemma that results in episodic weakness of the extremities [1]. Periodic paralysis can be classified into several types depending on the serum potassium level and etiology. Familial periodic paralysis, an autosomal dominant disease of the skeletal muscles, is the most common type of periodic paralysis in Western countries, whereas thyrotoxic periodic paralysis (TPP) and sporadic periodic paralysis (SPP) are the two major types in Asia [2].

TPP is a potentially lethal complication of hyperthyroidism characterized by muscle paralysis and hypokalemia due to massive intracellular shift of potassium [3]. TPP typically occurs a few hours after a heavy carbohydrate meal, alcohol consumption, or strenuous exercise [4]. However, the etiology and pathophysiology of TPP have not been fully elucidated.

Hyperthyroidism is a common condition with a global prevalence of 0.2–1.3% [5], and TPP was observed in 1.9% of patients with hyperthyroidism in a 1957 Japanese report [6]. Most cases of TPP are attributed to Graves’ disease [3]. According to a report of 46 patients with periodic paralysis admitted to a tertiary hospital in 1965 [7], 19 had TPP, 6 had familial hypokalemic periodic paralysis, and 17 had SPP. The underlying causes of TPP were not described in that report. It has been reported that the frequency of TPP has decreased in Japan, which may be attributed to the reduction in carbohydrate intake [8]. In contrast, the number of TPP cases reported in Western countries has increased recently [3].

Since TPP is a rare condition, only a few studies have elucidated the clinical characteristics of patients suffering from this disease. To achieve an early diagnosis of hyperthyroidism underlying TPP, we analyzed the recent clinical characteristics and backgrounds of patients with TPP who were emergently admitted to our hospital and compared them with those of the patients with thyrotoxicosis without periodic paralysis and SPP.

## Materials and methods

### Ethical considerations

The study protocol was reviewed and approved by the Institutional Review Board of the National Center for Global Health and Medicine (NCGM) [approval number: NCGM-S-004491-00]. The study was conducted following the tenets of the Declaration of Helsinki. This retrospective, non-interventional database study was conducted without patient involvement. According to the Guidelines for Epidemiological Studies formulated by the Ministry of Health, Labor, and Welfare of Japan, written informed consent was not required. Information regarding the study was available to the patients on the institutions’ website, and the patients had the right to cease registration of their data at any time.

### Study design and participant selection

This was a single-center, retrospective cohort study. Patients aged ≥18 years who were emergently admitted to NCGM and diagnosed with Graves’ disease, painless thyroiditis, subacute thyroiditis, or periodic paralysis between April 1, 2010, and March 31, 2022, were included in our study. This study only included patients admitted to the hospital on an emergency basis, and excluded those who received urgent treatment in the emergency room without requiring hospitalization. Patient data were retrieved from the electronic medical records. Thyroid diseases were diagnosed by the endocrinologists. Periodic paralysis was defined as the presentation of acute flaccid paralysis that spontaneously returned to normal with or without intervention. TPP was defined as periodic paralysis with elevated thyroid hormone and suppressed thyroid-stimulating hormone (TSH) levels. SPP was defined as periodic paralysis without any family history of periodic paralysis, hyperthyroidism, or other known causes of periodic paralysis.

In study 1, we compared the clinical backgrounds and laboratory data of patients with thyrotoxicosis who were emergently admitted with thyrotoxicosis-related symptoms with and without paralytic symptoms. In study 2, we compared the clinical backgrounds and laboratory data of patients with periodic paralysis with and without thyrotoxicosis.

Data collection was conducted from July 8, 2022, to March 31, 2023, after approval by the Institutional Review Board. Since the study involved collecting data from each patient’s electronic medical records, the authors had access to information that could identify individual participants during the study period.

### Clinical background and laboratory data

Clinical background information included age, sex, height, weight, body mass index (BMI), blood pressure (BP), heart rate (HR), smoking and drinking habits, history of thyrotoxicosis, comorbidities such as diabetes and atrial fibrillation, and family history of diabetes and thyroid disorders. Information regarding excessive exercise, high carbohydrate intake, and alcohol consumption as possible triggers for periodic paralysis was also collected. Laboratory data included electrolytes, venous blood gas, blood glucose, hemoglobin A1c (HbA1c), thyroid hormones, and thyroid-related antibodies.

Normalization of serum potassium level was defined as a value exceeding the lower limit (3.5 mEq/L) of the reference value through potassium supplementation. Recovery from paralysis was determined based on physical examination findings in the medical records. Information regarding the time to normalization of serum potassium level and amount of potassium supplementation until normalization were also collected.

### Statistical analysis

Data are expressed as mean ± standard deviation. Significant differences were tested using unpaired Student’s t-test or Mann–Whitney U test for continuous variables and Pearson’s Chi-square test or Fisher’s exact test for categorical variables. Spearman’s rank correlation coefficient was used to assess the correlation between variables. Statistical significance was set at P < 0.05. All the statistical analyses were performed using SPSS version 27 (IBM Co., Armonk, NY, USA).

## Results

### Study 1: Comparison of clinical data of patients with hyperthyroidism with and without periodic paralysis

**Fig 1:**
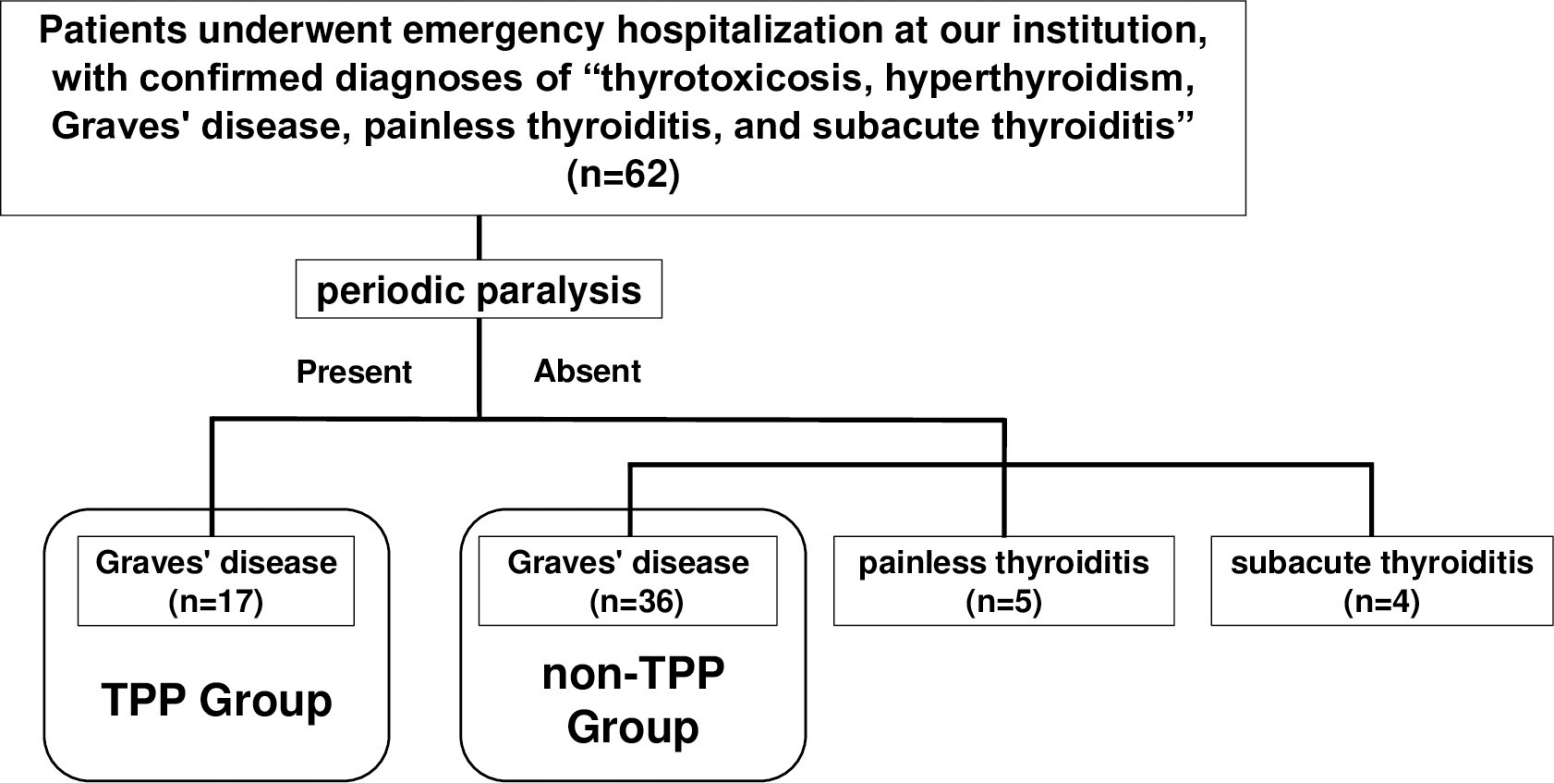
Flow diagram of study 1. TPP, thyrotoxic periodic paralysis.

During the study period, 62 patients with confirmed diagnoses of "thyrotoxicosis, hyperthyroidism, Graves’ disease, painless thyroiditis, or subacute thyroiditis” were admitted on an emergent basis. Individual verification of electronic medical records revealed that 17 of these patients had TPP (TPP group), and the cause of hyperthyroidism was Graves’ disease in all cases (Fig 1). Fifteen patients in the TPP group were men. Of the 17 patients with TPP, 14 were diagnosed with Graves’ disease for the first time (Table 1), two withdrew from treatment for Graves’ disease, and one had been treated for Graves’ disease but still had thyrotoxicosis. The average serum potassium level on admission was 2.3±0.75 mEq/L. No significant correlation was found between thyroid hormone levels and serum potassium levels or the amount of potassium required for normalization. Although no significant correlation was observed between serum potassium level and the amount of potassium required for normalization, a positive correlation was observed between age and the amount of potassium required for normalization (S1 Fig).

**Table 1:** Comparison of clinical features and laboratory data of TPP and non-TPP groups.

	TPP group (n = 17)	Non-TPP group (n = 36)	*P*
Age (years)	36.4 ± 12.3	52.7 ± 17.4	0.002*
Sex, male (%)	15 (88.2%)	10 (27.8%)	<0.001*
Body mass index (kg/m^2^)	24.2 ± 4.95	20.7 ± 4.26	0.014*
Systolic blood pressure (mmHg)	151 ± 18.9	138 ± 30.8	0.060
Diastolic blood pressure (mmHg)	77.8 ± 10.1	80.6 ± 24.3	0.817
Heart rate (bpm)	102.1 ± 17.8	125.8 ± 33.1	0.013*
Diagnosed with Graves’ disease for the first time n (%)	14 (82.4%)	25 (69.5%)	0.506
TSH (μIU/mL)	0.004 ± 0.005	0.002 ± 0.004	0.462
FT3 (pg/mL)	14.2 ± 8.24	15.9 ± 9.49	0.668
FT4 (ng/mL)	4.61 ± 2.06	5.83 ± 2.91	0.097
TRAb (IU/L)	17.5 ± 29.7	25.9 ± 34.1	0.086
Serum sodium (mEq/L)	143.1 ± 2.00	140.2 ± 6.36	0.071
Serum potassium (mEq/L)	2.26 ± 0.75	4.05 ± 0.58	<0.001*
Serum chlorine (mEq/L)	106.8 ± 3.36	104.7 ± 6.44	0.244
Serum calcium (mg/dL)	9.23 ± 0.60	9.55 ± 0.70	0.094
Serum phosphorus (mg/dL)	2.44 ± 0.74	3.94 ± 1.01	0.001*
Serum magnesium (mg/dL)	1.77 ± 0.18	2.40 ± 1.74	0.218

Abbreviations: TPP, thyrotoxic periodic paralysis; Non-TPP, thyrotoxicosis without periodic paralysis; TSH, thyroid stimulating hormone; FT4, free thyroxine; FT3, free triiodothyronine; TRAb; TSH receptor antibody.

Data presented as mean ± standard deviation or n (%).

*P < 0.05.

Among the 45 patients with thyrotoxicosis without paralysis, 36 had Graves’ disease, four had subacute thyroiditis, and five had painless thyroiditis. Symptoms for emergency hospitalization included palpitations, gastrointestinal symptoms, respiratory distress, edema, dizziness, and tremors; these symptoms were confirmed to be caused by thyrotoxicosis in all cases. Since all patients in the TPP group had Graves’ disease, we compared the data of patients in the TPP group and those with Graves’ disease without TPP (non-TPP group, n = 36). Of the 36 patients in the non-TPP group, 27 were diagnosed with Graves’ disease for the first time. The TPP group demonstrated significantly younger age (mean age: 36.4±12.3 years in the TPP group, 52.7±17.4 years in the non-TPP group, P < 0.05), higher proportion of male patients (males: 88.2% in the TPP group, 27.8% in the non-TPP group, P < 0.001), higher BMI, lower serum potassium and phosphorus level compared with the non-TPP group. Family history was compared in patients for whom information was available, and the TPP group had a higher rate of family history of diabetes than the non-TPP group (77.8% in the TPP group, 31.6% in the non-TPP group, P < 0.05).

### Study 2: Comparison of clinical data of patients with periodic paralysis with and without hyperthyroidism

During the same period as study 1, 92 patients were emergently admitted with a confirmed or suspected diagnosis of "periodic paralysis" or "hypokalemic periodic paralysis.” Of these, 41 patients were excluded from the analysis because their periodic paralysis was not confirmed in their medical records. Additionally, eight more patients were excluded from the analysis due to missing thyroid hormone level data. Of the remaining 43 patients, the TPP group consisted of the same 17 patients with Graves’ disease as in Study 1.

**Fig 2:**
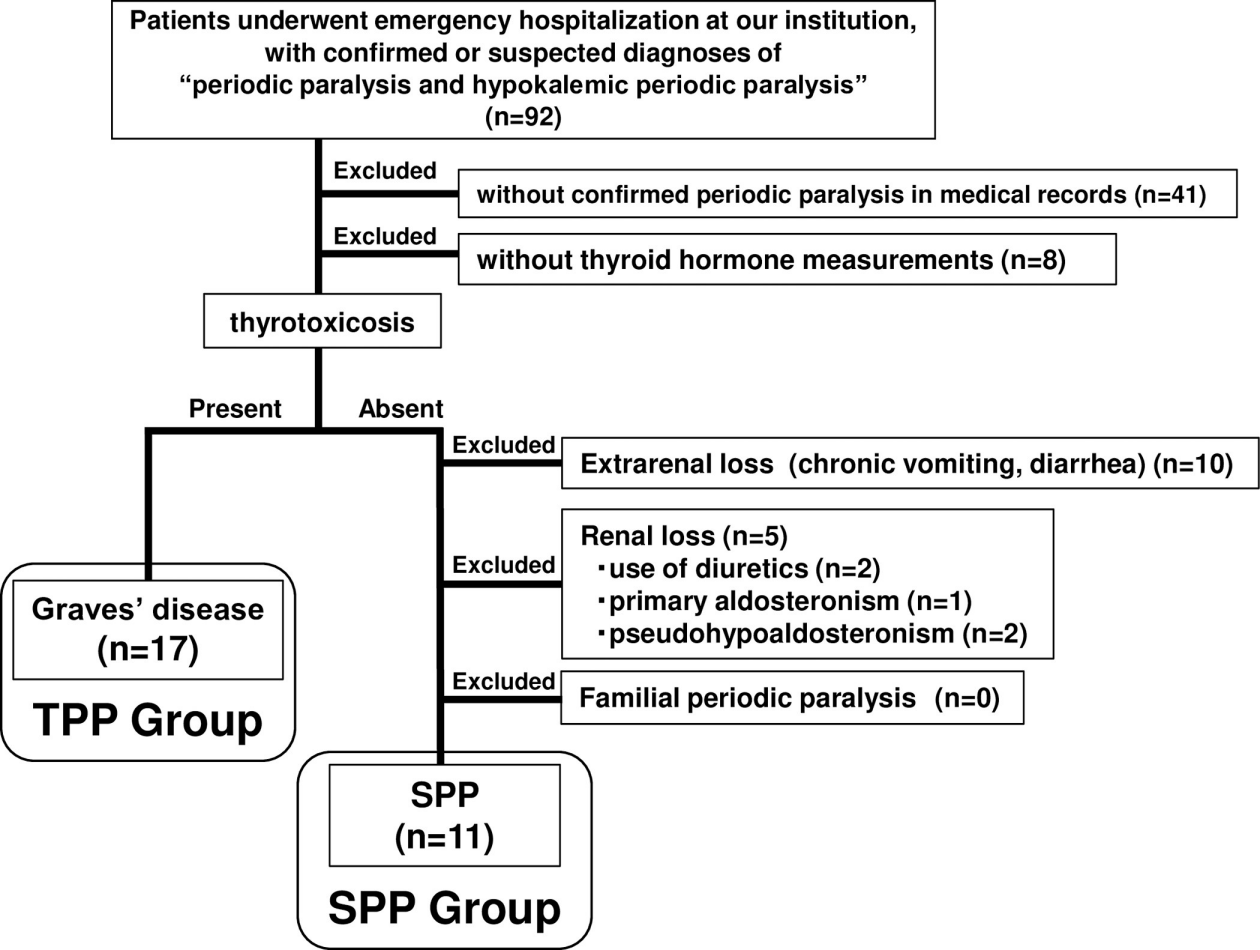
Flow diagram of study 2. TPP; thyrotoxic periodic paralysis, SPP; sporadic periodic paralysis.

Of the 26 patients with normal thyroid function, those with an obvious cause of hypokalemia were excluded: 10 patients demonstrated extrarenal potassium loss (chronic vomiting or diarrhea), and five exhibited renal potassium loss (two diuretic users, one with primary hyperaldosteronism, and two with pseudo hyperaldosteronism). No patient had familial periodic paralysis. Consequently, 11 euthyroid patients with periodic paralysis were classified into the SPP group (Fig 2), and their clinical findings were compared with those of the TPP group.

All patients with SPP were males. The TPP group demonstrated a significantly higher HR. No significant differences were observed between the SPP and TPP groups in terms of age, sex, BP, or serum electrolyte levels at admission (Table 2). No significant differences were observed between the two groups in the context of the affected limbs (S1 Table). Although there was no significant difference between the two groups, quadriplegia and bilateral lower-limb paralysis were equally common in the TPP group, whereas quadriplegia was more prevalent in the SPP group (77.8%).

**Table 2:** Comparison of clinical features and laboratory data of TPP and SPP groups.

	TPP group (n = 17)	SPP group (n = 11)	*P*
Age (years)	36.4 ± 12.3	41.7 ± 15.5	0.547
Sex, male (%)	15 (88.2%)	11 (100%)	0.505
Body mass index (kg/m^2^)	24.2 ± 4.95	26.1 ± 6.72	0.429
Systolic blood pressure (mmHg)	151 ± 18.9	143 ± 19.4	0.073
Diastolic blood pressure (mmHg)	77.8 ± 10.1	76.3 ± 8.48	0.689
Heart rate (bpm)	102.1 ± 17.8	83.6 ± 16.0	0.008*
TSH (μIU/mL)	0.004 ± 0.005	1.75 ± 1.14	<0.001*
FT3 (pg/mL)	14.2 ± 8.24	3.05 ± 0.55	<0.001*
FT4 (ng/mL)	4.61 ± 2.06	1.15 ± 0.21	<0.001*
Serum sodium (mEq/L)	143.1 ± 2.00	143.9 ± 3.27	0.926
Serum potassium (mEq/L)	2.26 ± 0.75	2.38 ± 0.53	0.640
Serum chlorine (mEq/L)	106.8 ± 3.36	107.5 ± 2.87	0.564
Serum calcium (mg/dL)	9.23 ± 0.60	8.68 ± 0.40	0.104
Serum phosphorus (mg/dL)	2.44 ± 0.74	2.30 ± 0.41	0.808
Serum magnesium (mg/dL)	1.77 ± 0.18	1.82 ± 0.26	0.335
potential Hydrogen	7.39 ± 0.06	7.39 ± 0.05	0.917
Bicarbonate ion (mmol/L)	26.6 ± 3.18	25.2 ± 4.71	0.365
The amount of potassium required for normalization (mEq)	74.2 ± 61.6	82.0 ± 63.2	0.750

Abbreviations: TPP, thyrotoxic periodic paralysis; SPP, sporadic periodic paralysis; TSH, thyroid stimulating hormone; FT4, free thyroxine; FT3, free triiodothyronine.

Data presented as mean ± standard deviation or n (%).

*P < 0.05.

Family history and triggering factors for paralysis were compared among patients for whom information was available (S1 Table). The prevalence of family history of diabetes was higher in the TPP group than in the SPP group (TPP group, 77.6%; SPP group, 0%; P <0.05). No significant differences were observed in the triggering factors between the two groups. Excessive exercise was observed in 3/14 (21.4%) patients in the TPP group and in 1/7 (7.0%) patient in the SPP group. High carbohydrate intake was evident in 3/16 (18.8%) and 3/10 (30.0%) patients in the TPP and SPP groups, respectively. Alcohol consumption was elucidated in 2/16 (12.5%) patients in the TPP group and 3/10 (30.0%) patients in the SPP group. Further, 10 of 17 (58.8%) patients in the TPP group and 5/11 (45.5%) in the SPP group did not have these provoking factors. There were no significant differences in the amount of potassium supplementation required for normalization of serum potassium level or time taken for symptom recovery.

## Discussion

In this study, we demonstrated the latest clinical features of TPP by analyzing 92 emergently hospitalized patients with thyrotoxicosis and/or periodic paralysis. The TPP group showed significant differences in terms of age, sex, and BMI, as well as a higher prevalence of hypokalemia and hypophosphatemia compared with the non-TPP group. These findings are consistent with the results of a previous study involving 15 patients with TPP in India [9]. Thyroid hormone levels were significantly lower in patients with TPP than those in non-TPP in the aforementioned Indian study; however, we did not observe any significant difference between the two groups. Non-TPP patients who were admitted in the emergency may have exhibited several biases, including age and underlying medical conditions (such as heart disease or lung disease). Therefore, the non-TPP group did not reflect the average characteristics of Graves’ disease. However, in emergency settings, our findings are considered useful for distinguishing between TPP and non-TPP patients.

When comparing the TPP and SPP groups, except for the significantly higher HR due to hyperthyroidism in the TPP group, no significant differences were observed between the two groups in terms of age, sex, BMI, or serum electrolyte levels at admission. These findings were similar to reports from Taiwan in the 1990s and the 2000s [2,10]. In addition, our analysis revealed no significant differences in the amount of potassium supplementation required to normalize serum potassium levels and the time to recovery between the TPP and SPP groups. These findings suggest that it is difficult to distinguish between TPP and SPP based on general clinical parameters alone.

In our study, Graves’ disease was diagnosed after the onset of paralysis in 82% of patients with TPP. Since patients with TPP experience paralysis upon arrival at the hospital, the signs and symptoms of hyperthyroidism may not be obvious. Graves’ disease should be diagnosed before the onset of paralysis. The reasons for the lack of diagnosis were uncertain because information regarding the course of hyperthyroidism symptoms before the onset of paralysis was unavailable.

TPP attacks reportedly develop only when a patient is in a thyrotoxic state [4]. However, no significant correlation exists between free thyroxine (FT4) and pretreatment serum potassium levels in patients with TPP. Furthermore, we did not find significant correlation between FT4 or pretreatment serum potassium levels and the amount of potassium supplementation required for normalization. Based on these findings, thyroid hormone levels potentially do not exhibit any relationship with the degree of hypokalemia. These results suggest that mechanisms other than excess thyroid hormone levels are responsible for the onset of TPP.

Several aspects of the pathophysiology of TPP remain unclear. Some evidence indicates that TPP results from a combination of genetic susceptibility, thyrotoxicosis, and environmental factors such as a high carbohydrate diet [11]. Potassium ions in the body are regulated mainly by two ion channels: the sodium-potassium (Na-K) pump and the inward-rectifying potassium channel (Kir) [12]. These channels work together to maintain serum potassium levels. The Na-K pump transports potassium into the cells (influx), whereas Kir regulates the outward flow of potassium ions (efflux) [12]. A recent study showed that some patients with TPP had mutations in *KCNJ18*, which encodes Kir2.6 expressed in the skeletal muscles [13]. However, *KCNJ18* mutations have been identified in only 25% of Asian patients with TPP, suggesting the involvement of other mechanisms [13].

Recently, several genome-wide association studies (GWAS) of Chinese and Taiwanese populations have revealed that some single nucleotide variants (SNVs) are associated with both TPP and SPP [14,15], suggesting that TPP and SPP may share a pathogenic mechanism independent of thyroid hormones [14]. Previous studies have reported the influence of catecholamines [4], insulin [4,16–19], androgens [4,20–22] and other triggering factors [4,23] on the pathophysiology of TPP. In our study, none of the patients with TPP had diabetes; however, the number of patients with a family history of diabetes was significantly higher in the TPP group than in the non-TPP and SPP groups. Unfortunately, our study did not include an assessment of serum insulin level and glucose tolerance. Previous studies have established a link between a family history of diabetes and insulin resistance and secretion [24,25]. Other studies have suggested that hyperinsulinemia precedes TPP attacks [26] and that patients with TPP exhibit a higher prevalence of obesity and lower insulin sensitivity than those with simple thyrotoxicosis [19]. These results suggest that not only thyrotoxicosis but also a predisposition to hyperinsulinemia might influence TPP. No significant difference was observed in the prevalence of other triggering factors between the TPP and SPP groups. A previous report [23] also showed that 66% of the TPP cases had no apparent triggers such as excessive carbohydrate intake or physical exertion. Further investigation is required to gain a comprehensive understanding of periodic paralysis.

The clinical characteristics of TPP identified in this study suggest that it is challenging to distinguish between TPP and SPP based on the clinical information at admission and clinical course. TPP may be overlooked if the treatment is completed with potassium supplementation alone in patients hospitalized due to periodic paralysis. Therefore, when treating patients with hypokalemic periodic paralysis due to intracellular potassium shift, measurement of thyroid hormone levels is necessary in all cases.

Our study had a few limitations that need consideration. This was a single-center, retrospective investigation. Although our facility is designated as a tertiary emergency hospital and has accumulated a considerable number of emergency patients, the sample size was relatively small. In addition, we were unable to conduct urine electrolyte tests upon admission in most cases.

In conclusion, patients with TPP were predominantly young males, and the cause of hyperthyroidism in all cases was Graves’ disease. These characteristics have remained consistent over the past two decades. Patients with periodic paralysis typically recover early with appropriate treatment, including transvenous potassium replacement. However, for the early detection and launch of specific treatment of Graves’ disease, not overlooking the hyperthyroidism underlying the periodic paralysis is crucial. Considering that most of the underlying diseases of TPP are undiagnosed Graves’ disease, distinguishing between TPP and SPP based on clinical information at admission and clinical course alone is challenging in emergency settings. Therefore, screening for thyroid hormone and anti-thyroid stimulating hormone receptor antibody levels is necessary when treating patients with periodic paralysis.

## Supporting information

S1 Fig Correlation between serum potassium level and serum FT4 level (A), the amount of potassium required for normalization and serum FT4 level (B), serum potassium level (C), and age (D). *P < 0.05. FT4, free thyroxine.
(TIF)

S1 Table Comparison of family history and triggering factors of TPP and SPP groups. Abbreviations: TPP, thyrotoxic periodic paralysis; SPP, sporadic periodic paralysis. Data presented as mean ± standard deviation or n /N (%). *P < 0.05.
(DOCX)
