# Supplementary figures and images for: A single-center retrospective study on the clinical features of thyrotoxic periodic paralysis
Source: PLoS One. 2024 Aug 1;19(8):e0308076. doi: 10.1371/journal.pone.0308076 (PMC11293632; doi:10.1371/journal.pone.0308076)

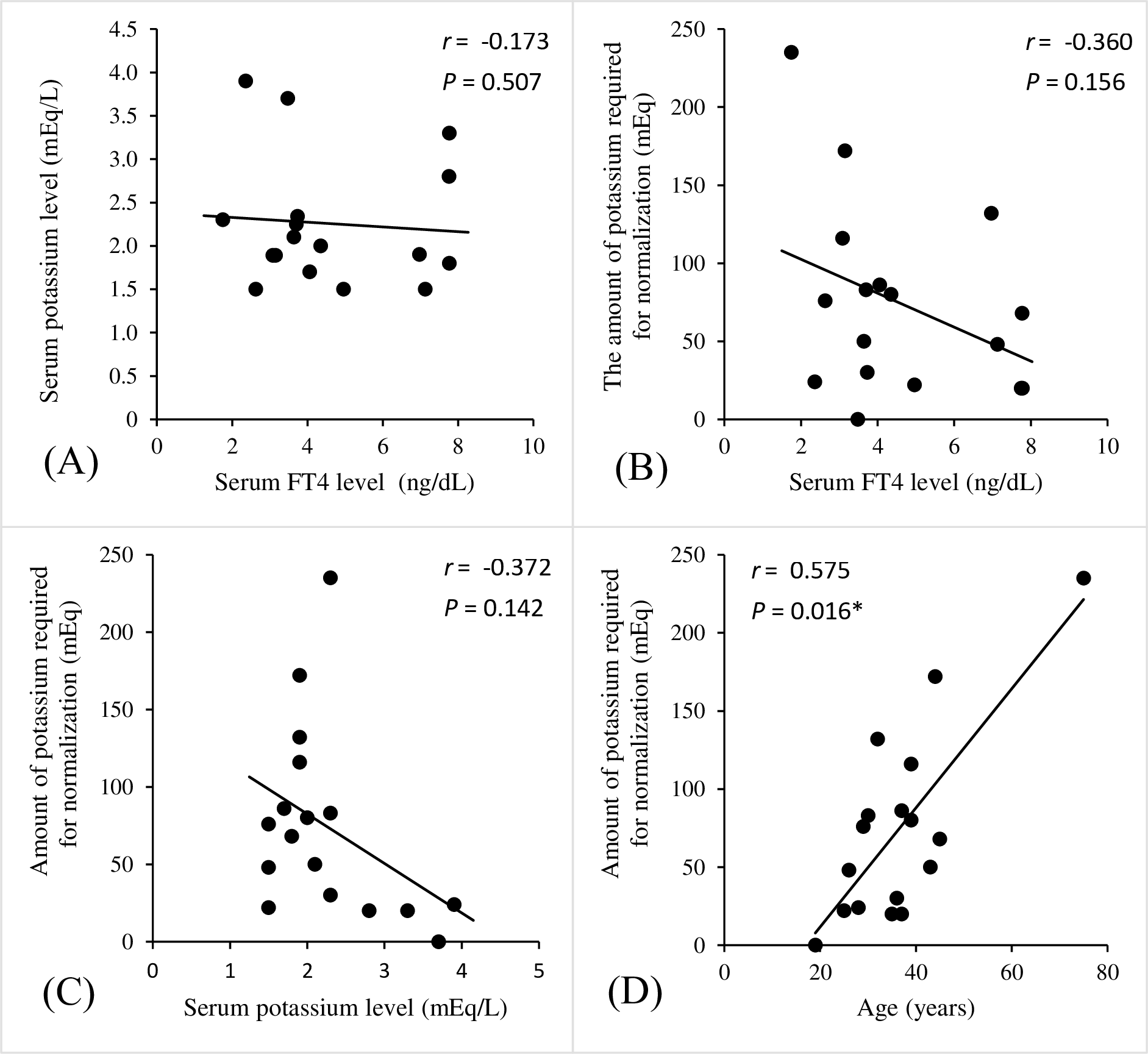

Supplement: S1 Fig — Correlation between serum potassium level and serum FT4 level (A), the amount of potassium required for normalization and serum FT4 level (B), serum potassium level (C), and age (D). *P < 0.05. FT4, free thyroxine. (TIF) [file pone.0308076.s001.tif]
