# Supplementary material for: A single-center retrospective study on the clinical features of thyrotoxic periodic paralysis
Source: PLoS One. 2024 Aug 1;19(8):e0308076. doi: 10.1371/journal.pone.0308076 (PMC11293632; doi:10.1371/journal.pone.0308076)
Supplement: S1 Table — Abbreviations: TPP, thyrotoxic periodic paralysis; SPP, sporadic periodic paralysis. Data presented as mean ± standard deviation or n /N (%). *P < 0.05. (DOCX) [file pone.0308076.s002.docx]

**S Table 1. Comparison of family history and triggering factors of TPP and SPP groups.**

|  | | TPP group  (n=17) | SPP group  (n=11) | *P* |
| --- | --- | --- | --- | --- |
| Affected limbs | Quadriplegia (%) | 8/17 (47.1%) | 7/9 (77.8%) | 0.022 |
|  | Bilateral lower-limb paralysis (%) | 9/17 (52.9%) | 2/9 (22.2%) |  |
| Family history of diabetes (%) | | 7/9 (77.8%) | 0/5 (0%) | 0.025* |
| Triggering factors | Excessive exercise (%) | 3/14 (21.4%) | 1/7 (7.0%) | 0.543 |
|  | High carbohydrate intake (%) | 3/16 (18.8%) | 3/10 (30.0%) | 0.824 |
|  | Alcohol consumption (%) | 2/16 (12.5%) | 3/10 (30.0%) | 0.537 |
|  | Without those triggering factors (%) | 10/17 (58.8%) | 5/11 (45.5%) | 0.700 |
